# Uterine Sarcoidosis: A Rare Extrapulmonary Site of Sarcoidosis

**DOI:** 10.1155/2013/706738

**Published:** 2013-05-20

**Authors:** Creticus P. Marak, Narendrakumar Alappan, Amit Chopra, Olena Dorokhova, Sumita Sinha, Achuta K. Guddati

**Affiliations:** ^1^Division of Pulmonary and Critical Care Medicine, Montefiore Hospital, Albert Einstein College of Medicine, Yeshiva University, New York, NY 10467, USA; ^2^Department of Pathology, Montefiore Hospital, Albert Einstein College of Medicine, Yeshiva University, New York, NY 10467, USA; ^3^Department of Internal Medicine, Massachusetts General Hospital, Harvard Medical School, Harvard University, Boston, MA 02014, USA

## Abstract

Sarcoidosis is a multisystem disease which is most commonly manifested in the pulmonary system. However, extrapulmonary manifestations have also been frequently reported. Isolated occurrence of sarcoidosis in the genital system is rare and poses a diagnostic and therapeutic dilemma. Uterine sarcoidosis can present with cervical erosions, endometrial polypoid lesions, and recurrent serometra. In majority of cases, it is diagnosed by endometrial curettage, but it has also been detected by examination of hysterectomy, polypectomy, and autopsy specimens. Nonnecrotizing granulomas are the characteristic pathologic finding of sarcoidosis. However, many infectious and noninfectious etiologies including certain neoplasms can produce similar granulomatous reactions in the female genital tract. These conditions affect the female genital tract more commonly than sarcoidosis, and therefore it is important to rule out these conditions first before making a diagnosis of sarcoidosis. Treatment of sarcoidosis is different from treating these other conditions and the most commonly used systemic or local corticosteroids can be hazardous if the underlying cause is infection. In this case report, the clinical presentation, histopathology, clinical course, and treatment of a patient with isolated uterine sarcoidosis are described, and a brief literature review of sarcoidosis of the female genital tract is provided.

## 1. Introduction

Sarcoidosis is a multisystem disease of unclear etiology. Any organ system in the body can be affected by sarcoidosis, and the classic pathologic finding is the presence of noncaseating granulomas in the involved organs. Pulmonary sarcoidosis is the most common manifestation of the disease, accounting for 90 percent of the cases [1]. Extra pulmonary sarcoidosis is also common, with skin, eyes, liver, and reticuloendothelial manifestations accounting for the majority of the cases (10–25%). The involvement of other organ systems such as cardiovascular, nervous system, upper respiratory tract, renal, spleen, thyroid, gastrointestinal tract, musculoskeletal, and exocrine glands accounts for only a minority of cases (0.4–5%) [1–4]. Most of the cases with extra pulmonary sarcoidosis have coexisting pulmonary disease. This was shown by ACCESS research group where 368/736 (52%) of their patients had concomitant pulmonary involvement and only 14/736 (1.9%) of their patients had isolated extra pulmonary sarcoidosis [5]. Involvement of the female reproductive system by sarcoidosis is very rare. Unlike other extra pulmonary manifestations, there is not much data available for this variant of sarcoidosis; whatever little information we have is mostly based on case reports.

## 2. Case Description

**Figure 1:**
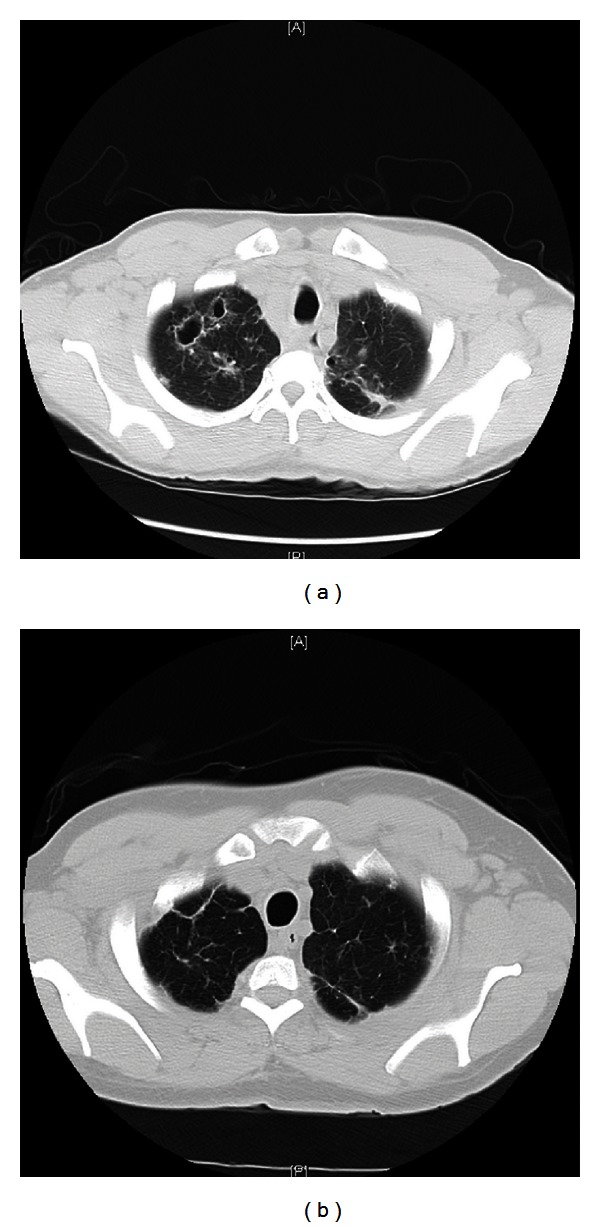
(a) CT scan of chest revealing cavitary lesions. (b) CT scan of chest shows resolution of cavitary lesions.

**Figure 2:**
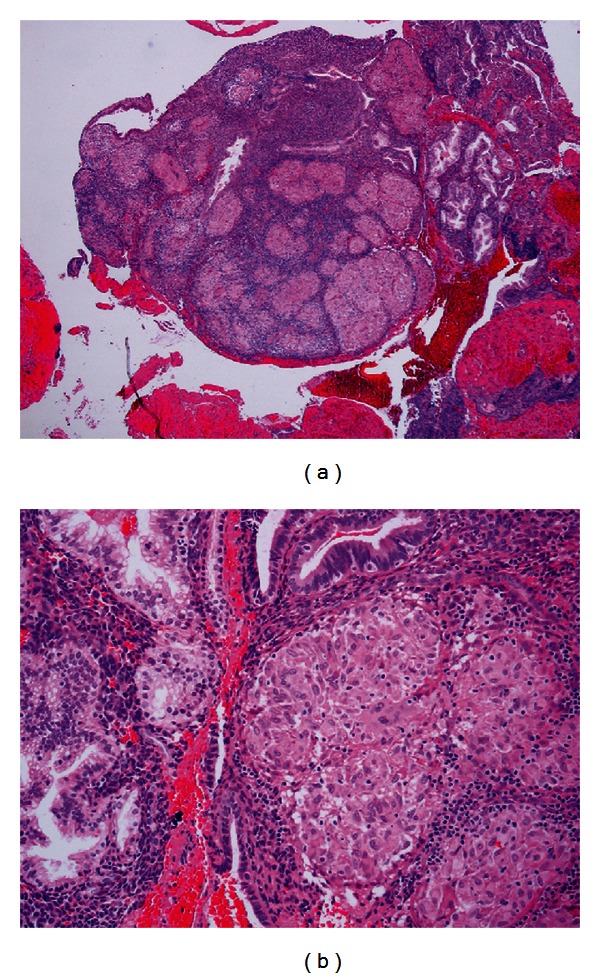
(a) Hematoxylin and eosin (H&E) stain at low magnification shows noncaseating granulomas. (b) H&E stain at high magnification shows a follicular pattern of granulomas, asteroid bodies, and the absence of necrosis.

A 45-year-old African American premenopausal female, gravida 1 and para 1, chronic active smoker (20 pack years), and with a past medical history significant for schizophrenia and chronic obstructive pulmonary disease presented to our facility with menorrhagia. She was recently treated for latent syphilis with penicillin, and she also has genital warts and herpes virus 2 infections. Three years prior, she presented with mediastinal adenopathy and thick walled pulmonary cavities involving both the upper lobes. Flexible bronchoscopy with bronchoalveolar lavage and transbronchial biopsies from the right upper lobe revealed nonnecrotizing granulomas consistent with sarcoidosis. They were negative for AFB and fungal stains. A diagnosis of primary cavitary pulmonary sarcoidosis was made, and she was treated with systemic steroids for twelve months. She responded well to steroids with complete clinical and radiographic resolutions (Figures 1(a) and 1(b)). Fourteen months back she presented with epigastric and right upper quadrant pain of a one-month duration. Esophagogastroduodenoscopy revealed a clean gastric ulcer along the lesser curvature and a markedly erythematous and edematous antrum. Biopsies from the gastric antrum revealed features of chronic gastritis and nonnecrotizing granulomas which was consistent with her previous diagnosis of sarcoidosis. Her symptoms resolved with proton pump inhibitors, and she did not require additional treatment with steroids for her gastric sarcoidosis. In this admission, she presented with menorrhagia of two-month duration. At her baseline, her menstrual cycles are regular and last for 3-4 days. Her vitals and systemic examinations were unremarkable. Pelvic examination revealed multiple subcentimeter white ulcerated lesions involving the labia majora. Her hemoglobin level was 9.5 gm/dL and the rest of her routine laboratory workup including complete blood count, coagulation profile, liver, and renal function tests was normal. She tested negative for HIV. Chest roentgenogram did not reveal any abnormality. On transvaginal ultrasonography, size of the uterus was 12.1 × 6.5 × 8.2 cm^3^ with an endometrial thickness of 6 mm. It also revealed an anterior body mural fibroid with a submucosal component measuring 4.3 × 3.7 × 4.4 cm^3^ and a fundal fibroid measuring 2.9 × 2.7 × 2.9 cm^3^; her ovaries were normal. She received a short course of high dose oral contraceptive pills. Punch biopsies from the vulvar lesions revealed high grade squamous intraepithelial lesions with features of human papillomavirus infection. Therapeutic endometrial curettage was performed, and the histopathological examination of the endometrial tissues revealed menstrual phase changes, strands of smooth muscles suggestive of uterine myoma, and numerous nonnecrotizing epithelioid giant cell granulomas (Figures 2(a) and 2(b)). Stains and cultures for bacteria, fungal, and mycobacteria were negative, and she was diagnosed with uterine sarcoidosis. She has been amenorrhoeic and asymptomatic since she underwent a therapeutic endometrial curettage.

## 3. Discussion

Sarcoidosis of the female genital tract is probably the rarest form of all the extra pulmonary sarcoidosis, accounting for less than 1% of cases [6]. Based on the literature review, it seems that the uterus is the most commonly involved organ of the female genital tract [7, 8]. In 1933, Garland and Thompson first described an autopsy finding of sarcoidosis involving the uterus in a 28-year-old female. Since its first description in 1933 till 1988, only 21 cases of female genital tract sarcoidosis were reported in the literature [6]. In the following years, there have been just a few more reported cases of uterine sarcoidosis [9–18]. Most of the cases occur in the reproductive age (21–40 years), although a case of uterine sarcoidosis in a 64-year-old postmenopausal woman has also been described [19]. Like any other extra pulmonary manifestation of sarcoidosis, most of the patients have concomitant pulmonary involvement and the disease seems to occur more frequently in the Black population. A majority of the patients present with menstrual irregularities; menorrhagia, metrorrhagia, and postmenopausal bleeding are more commonly reported symptoms than amenorrhea [6, 8–17]. Some of the patients were asymptomatic, and the diagnosis was made based on examination of the hysterectomy or autopsy specimens [6]. Some cases of uterine sarcoidosis presented with cervical erosions [8], endometrial polypoid lesions [18], and recurrent serometra [16]. Others were incidental findings on cervical pap smear [17] and patients who underwent surgery for uterine myomas. Endometrial curettage led to the diagnosis in the majority of the cases; in others the diagnosis was made on examination of the hysterectomy, polypectomy, and autopsy specimens [6, 17, 18]. Sampling bias probably accounts for the increased reporting of uterine sarcoidosis compared to other parts of the female genital tract [8]. Cases of uterine sarcoidosis with concomitant involvement of the ovaries and fallopian tubes have also been reported [6, 13]. Isolated cases of uterine sarcoidosis are usually self-limiting and have good prognosis; there seems to be no detrimental effect on pregnancy outcomes despite the presence of granulomas in the placenta. A majority of the patients do not require treatment and can be monitored; however, systemic steroids may be helpful in symptomatic patients [8]. 

Ovarian sarcoidosis is probably the second most common manifestation of female genital tract sarcoidosis. Winslow and Funkhouser in 1968 described a case of a 28-year-old female who underwent total abdominal hysterectomy with bilateral salpingoophorectomy for a cervical lesion [20]. Histopathological examination revealed sarcoid-like lesions involving the uterus, right fallopian tube, and the right ovary. Since then there have been other reports of ovarian involvement by sarcoidosis. Like uterine sarcoidosis, most of the reported cases occurred in the reproductive age group [7, 20–25], although a few cases of ovarian sarcoidosis in postmenopausal women have also been reported [26–28]. Symptoms can be nonspecific, such as fever, malaise, and abdominal pain [29], or they can present with clinical features concerning for ovarian tumors, such as ovarian enlargement, weight loss, obstructive uropathy, intraperitoneal mass, and ascites associated with thickening of the omentum and peritoneal nodular deposits [7, 21–24, 28]. In these cases, diagnosis was made based on histopathological findings and ruling out other causes of granulomatous inflammation. Some of these patients had elevated CA-125 levels, which are typically elevated in patients with ovarian tumors and carcinoma of the female genital tract [22–24, 28]. CA 125 is elevated also in various nongynecologic malignancies and other benign conditions as well [24]. CA 125 elevation is probably from increased production by epithelioid cells present in the granulomas or is due to increased production by peritoneal cells in response to inflammatory mediators secreted by sarcoid granulomas [24]. There have been reports of ovarian tumors with sarcoid-like lesions involving the bone marrow and regional lymph nodes [29–31]. These sarcoid-like reactions occur in other malignancies, such as lymphomas, breast cancer, primary lung cancer, renal cell, and gastric cancers. Usually these reactions are limited in distribution mostly involving the regional lymph nodes, but multiorgan involvement consistent with systemic sarcoidosis can develop simultaneously or following chemotherapy [29–35]. Finding a sarcoid-like lesion calls for careful examination for other adjacent lesions that may be masking malignancy. This is probably due to dysregulation in the Th1/Th2 immunity in these situations, and sarcoidosis in itself has been shown to have enhancement of the Th1 immunity [36, 37]. Careful examination of the pathology specimens is therefore indicated as these granulomatous lesions can easily be confused with metastatic disease and can cause treatment dilemma. Most of these patients tend to be symptomatic and have a good response to steroids with resolution of enlarged ovaries, intrapelvic mass, ascites, and normalization of CA 125 levels [7, 22–24, 28]. 

Involvement of the fallopian tubes commonly occurs in conjunction with sarcoidosis of the other parts of female genital tract. In 1954, Cowdell described an autopsy finding of sarcoidosis involving the fallopian tubes of a 21-year-old female who died of cardiac sarcoidosis [6, 38]. Subsequently more cases of tubal sarcoidosis have been described but the diagnosis was made mostly on pathologic examination of salpingectomy specimens in patients who were being treated for other gynecological problems. Most of these patients had menstrual irregularities, some had fibroids [6], and one of the patients had dyspareunia and infertility [38]. At the time of surgery distension of the tubes, pelvic adhesions and miliary deposits of sarcoid granulomas may be seen involving the tubes, uterus, ovaries, ligaments, omentum, and intestines [6, 7, 21, 22, 38]. Vaginal sarcoidosis is the rarest of all, and only two cases have been reported to date [39, 40]. One of the cases had pulmonary sarcoidosis who presented with vaginal itching and irritation and responded to systemic and topical steroids. Vulval sarcoidosis is another rare manifestation of sarcoidosis with only five cases reported to date [41–45]. One of the patients presented with papular rash involving the labia majora and the perineum; the second one presented with a painful nodular lesion at the site of the previous episiotomy site, and the third one presented with vulvar mass. Two of the patients had pulmonary sarcoidosis. Details on the other two are not available. 

Nonnecrotizing granulomas are the characteristic pathologic finding of sarcoidosis. However, this finding is not pathognomic of sarcoidosis and many infectious and noninfectious etiologies including certain neoplasms can produce similar granulomatous reactions in the female genital tract. Infectious etiologies include tuberculosis, atypical mycobacteria, endemic mycosis, actinomycosis, and parasites. Noninfectious causes like foreign body reaction, Crohn's disease, medications, lymphoma, and other neoplastic conditions and postprocedure or postsurgical granulomas can also mimic sarcoid granulomas [46–48]. These conditions affect the female genital tract more commonly than sarcoidosis, and therefore it is important to rule out these conditions first before prematurely making a diagnosis of sarcoidosis. Treatment of sarcoidosis is different from treating these other conditions and the most commonly used systemic or local corticosteroids can be hazardous if the underlying cause is infection. 

In conclusion, female genital tract sarcoidosis is rare and its presentation can mimic other conditions that commonly affect the female genital tract. Diagnosis is mainly based on identification of noncaseating granulomas in the involved organs and ruling out other conditions that can have a similar histopathology finding. It can affect any age group but usually occurs in the reproductive age group. Like other extra pulmonary manifestations of sarcoidosis, the majority of the patients have pulmonary involvement and the Black population seems to be affected more frequently than other races. Increased incidence of uterine sarcoidosis may just reflect a sampling bias as most of these cases had concomitant involvement of other structures of the female genital tract. Endometrial curettage or even pap smear may be sufficient to make the diagnosis of uterine sarcoidosis; involvement of other structures will need a biopsy. Isolated cases may just require observation but systemic and symptomatic patients may be given a trial of systemic steroids.
